# Bioinformatics Analysis and Structure of Gastric Cancer Prognosis Model Based on Lipid Metabolism and Immune Microenvironment

**DOI:** 10.3390/genes13091581

**Published:** 2022-09-03

**Authors:** Yongzhi Chen, Hongjun Yuan, Qian Yu, Jianyu Pang, Miaomiao Sheng, Wenru Tang

**Affiliations:** Laboratory of Molecular Genetics of Aging & Tumor, Medicine School, Kunming University of Science and Technology, Kunming 650032, China

**Keywords:** gastric cancer, immune microenvironment, lipid metabolism, targeted therapy, gastrointestinal cancers

## Abstract

Objectives: The reprogramming of lipid metabolism is a new trait of cancers. However, the role of lipid metabolism in the tumor immune microenvironment (TIME) and the prognosis of gastric cancer remains unclear. Methods: Consensus clustering was applied to identify novel subgroups. ESTIMATE, TIMER, and MCPcounter algorithms were used to determine the TIME of the subgroups. The underlying mechanisms were elucidated using functional analysis. The prognostic model was established using the LASSO algorithm and multivariate Cox regression analysis. Results: Three molecular subgroups with significantly different survival were identified. The subgroup with relatively low lipid metabolic expression had a lower immune score and immune cells. The differentially expressed genes (DEGs) were concentrated in immune biological processes and cell migration via GO and KEGG analyses. GSEA analysis showed that the subgroups were mainly enriched in arachidonic acid metabolism. Gastric cancer survival can be predicted using risk models based on lipid metabolism genes. Conclusions: The TIME of gastric cancer patients is related to the expression of lipid metabolism genes and could be used to predict cancer prognosis accurately.

## 1. Introduction

Gastric cancer is one of the most common cancers in the world. Although the incidence has declined with technological advances, the prognosis remains poor [1]. Gastric cancer occurs at different rates in different world regions and ethnic groups. Despite advances in identification and treatment, stomach cancer has just a 20% 5-year survival rate. Based on histologic features, genotypes, and molecular phenotypes, the new gastric cancer classification system helps researchers understand the differences between subtypes and improves early identification, prevention, and treatment [2,3,4].

Growing evidence indicates that reprogramming of lipid metabolism plays a crucial role in cancer [5,6]. The specific mechanisms of lipid metabolism in gastric cancer are still unknown, and the therapeutic targets are still in the preclinical stage. Studies show that the typical characteristics of lipid metabolism in gastric cancer are increased lipid synthesis and up-regulated β-oxidation and oxidative decomposition of fatty acid [7]. In the immune microenvironment of gastric cancer, the phosphoinositide 3-kinase (PI3K)-protein kinase B (AKT)-mammalian target of rapamycin (mTOR) pathway is abnormally activated, which stimulates the abnormal proliferation of malignant tumors. This pathway is an important factor in the growth, metabolism, metastasis, and drug resistance of gastric cancer [8]. The PI3K-AKT-mTOR pathway mainly regulates the abnormal uptake of newly synthesized fat and exogenous lipids in gastric cancer by controlling sterol regulatory element-binding proteins (SREBPs) [9]. The interaction between altered lipid metabolism and the TIME can affect cancer by promoting inflammation through lipid stimulation of tumors, accelerating angiogenesis [10], and affecting stromal cells, allowing the immune system to escape [11]. Rapidly proliferating cancer cells require a lot of energy. When energy supply is insufficient, ATP produced by fatty acid oxidation is an important energy source for cancer cells. This forces tumor cells to adjust their metabolic profiles, directly leading to the malignant transformation of tumor cells and abnormal lipid accumulation in the TIME [12]. Adipocytes and free fatty acids in the hypoxic TIME benefit cancer proliferation, progression, invasion, and metastasis. However, the exact role of lipid metabolism reprogramming in tumor immune responses remains unclear. A comprehensive understanding of lipid metabolism functions in the TIME and its dual effects on immune responses are critical for mapping the details of tumor immunology and developing specific treatments for cancer patients.

The TIME was critical for tumor commencement and progression [13]. The microenvironment of tumor cells is transformed methodically by the secretion of various biological chemicals, empowering neighboring cells with the power to influence the progression of cancers [14].Tumor-infiltrating immune cells are the predominant non-tumor components in the TIME, and these have been proven to make a significant contribution to prognostication. As a result, TIME is crucial to the formation and progression of tumors, and evidence suggests that TIME is involved in the pathogenesis of gastric carcinoma [15]. Assessing the TIME of gastric cancer will assist in understanding the immunological status of tumor cells.

Immunotherapy has shown to be effective in various solid tumors in recent studies [16]. Immunotherapy is an alternate treatment option for cancer patients that employs a different strategy than targeting the tumor directly as per conventional cancer treatment. Because immunotherapy relies on immune responses to identify and eliminate tumor cells, there has been a surge in interest in learning more about the tumor immune response. Novel biomarkers related to the tumor immune response have been developed due to expanded research. These biomarkers may allow for novel techniques in improving healing effects and increasing immunotherapy’s potential impact [17]. Consequently, it is necessary to establish a risk classification strategy and identify critical genes for gastric cancer patients to receive individualized, targeted treatment. Furthermore, earlier research has shown that lipid metabolism genes have a substantial predictive value in ovarian carcinomas [18], renal cell carcinoma (RCC) [19], lung adenocarcinoma (LAUD) [20], pancreatic cancer [21], and hepatocellular carcinoma (HCC) [22]. Targeting lipid metabolism as a novel cancer therapy strategy has been proposed [23]. Nevertheless, the effect of lipid metabolism genes in gastric cancer has remained poorly understood.

Survival analysis methods have been commonly used to identify the importance of prognostic factors in studying gastric cancer. Survival research has established multivariate prediction models using clinical characteristics and related them via molecular pathways [24]. Identifying differential expression genes is crucial for the correct diagnosis of tumor characteristics, developing new therapies, and delineating tumor behavior for more precise results and prognosis prediction [25]. As an efficient method for assessing prognosis, risk models have been developed to explore the predictive value of genes associated with the TIME and energy metabolism in gastric cancer [26].

This research focuses on the influence of lipid metabolism genes on the TIME of gastric cancer patients. We obtained novel subtypes by consensus clustering and analyzed their immune status by ESTIMATE, TIMER, and MCPcounter algorithms. Then, we try to elucidate possible pathways through functional analysis. Then, a novel five lipid metabolism genes signature (*AKR1B1*, *MTF1*, *PLA2R1*, *GGPS1*, and *ETNPPL*) was constructed to predict clinical outcomes in gastric cancer, which are listed in Table 1. Furthermore, we developed a risk score model to assess the predictive significance of lipid metabolism genes in gastric cancer. Our findings could lead to fresh insights into the underlying molecular pathways of gastric cancer and offer new insights on gastric cancer targeting therapy.

## 2. Materials and Methods

### 2.1. Data Sources and Pre-Processing

RNA data and related clinical information of gastric carcinoma patients were collected from UCSC databases. RNA data from 407 patients with gastric cancer were included, and RNA data were normalized by log2(x + 1). The RNA expression data of 407 patients with gastric cancer were randomly allocated to a 3/4 training set and 1/4 validation set. After excluding the samples without patient survival time or survival status in clinical information, 167 gastric carcinoma patients were obtained from the training set, and 80 gastric carcinoma patients were obtained from the verification set. Datasets of 745 lipid metabolism genes were obtained from the Molecular Signatures Database.

### 2.2. Identification of Molecular Subtypes

**Figure 1:**
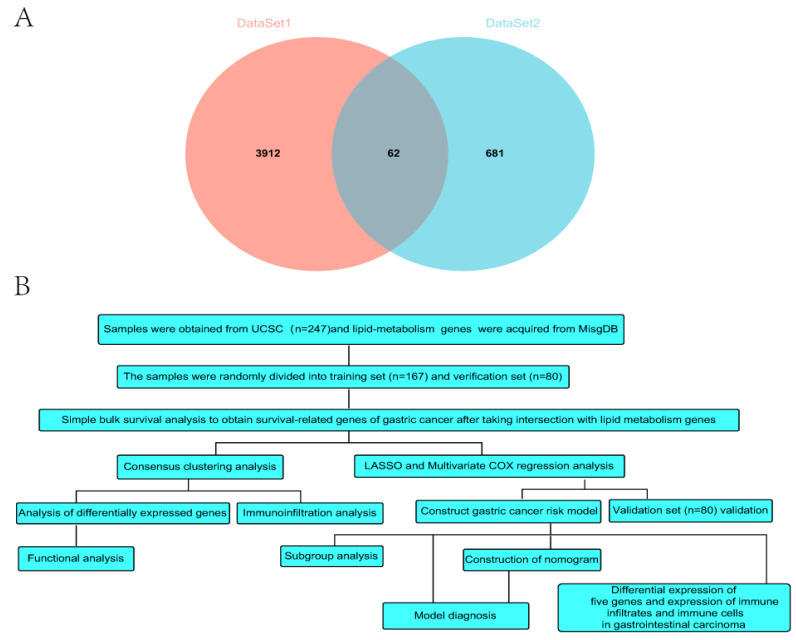
(**A**) Venn diagram of the intersection of survival genes and genes related to lipid metabolism. (**B**) Data analysis flow chart.

A total of 3972 genes (*p* < 0.05) were associated with gastric carcinoma prognosis through the simple batch survival analysis. A total of 62 genes were obtained by the intersection of 3972 genes (dataset1) related to gastric cancer survival and 745 genes (dataset2) related to lipid metabolism (Figure 1A). ConsensusClusterPlus [27] was employed to conduct cluster analysis based on the expression matrix of 62 genes associated with survival and lipid metabolism.

### 2.3. Immune Microenvironment Evaluation and Immune Cells Analyses

IOBR [28] is an immune tumor biology computing tool. Here, we select ESTIMATE [29], TIMER [30], and MCPcounter [31] algorithms, using the R package IOBR based on our expression spectrum. Expression data were used to obtain a stromal score, immune score, and ESTIMATE score. The TIMER algorithm calculated the abundance of six types of immune cells (B cell, macrophage cell, dendritic cell (DC), neutrophil cell, CD4 T cell, and CD8 T cell). Using the MCPcounter algorithm, we determined the abundance of ten immune cells and lineages.

### 2.4. Functional Analyses

Limma (Linear Models for Microarray Data) [32] is a differential expression screening method based on generalized linear models. For differential analysis, we used the R package limma to extract the differential genes. To enrich related pathways, we used Gene Ontology (GO), Kyoto Encyclopedia of Genes and Genomes (KEGG) analyses, and Gene Set Enrichment Analysis (GSEA) to determine the difference between clusters.

### 2.5. Development and Verification of Risk Model

This study integrated survival time, status, and gene expression data using the “glmnet” R software package. The lasso-Cox method was used for regression analysis. In addition, a ten-fold cross-validation was conducted to determine the optimal model. We selected a lambda minimum of 0.0466 and finally obtained 21 genes. Multivariate Cox regression analysis is applied to identify the genes used to establish the risk model. The risk score per each patient in the training and validation cohorts was calculated as: risk score = (0.284 * expression value of *AKR1B1*) + (0.235 * expression value of *ETNPPL*) + (0.437 * expression value of *GGPS1*) − (0.459 * expression value of *MTF1*) + (0.141 * expression value of *PLA2R1*). The patients were classified into a high-risk group and a low-risk group via the medium value. AUC was determined using the R software package pROC (Version 1.17.0.1) for receiver operating characteristic (ROC) analysis. Specifically, survival time and status of patients and gene expression values of *AKR1B1*, *MTF1*, *PLA2R1*, *GGPS1*, and *ETNPPL* were obtained. ROC analyses were performed at 365-day, 730-day, and 1095-day time points using pROC’s ROC function. The area under the curve (AUC) and confidence intervals were evaluated using pROC’s CI function to obtain the final AUC results. Then, we integrated survival time, survival status, and clinical data using the “rms” R software package to establish a nomogram by the Cox method and evaluate the prognostic significance of these dates.

### 2.6. Differential Expression and Immune Microenvironment of Five Genes in Gastrointestinal Cancers

We used the UCSC database to download a standardized universal cancer dataset. We also retrieved five gene expression data in various samples from TCGA Pan-cancer. In addition, we screened the sample sources as follows: primary blood-derived cancer and solid normal tissue. We also eliminated samples with a zero expression level and transformed each expression value using the log2(x + 0.001) transformation. We filtered cancers with less than three samples and obtained 26 cancers.

Finally, we chose four cancers: Cholangiocarcinoma (CHOL), Esophageal carcinoma (ESCA), Stomach adenocarcinoma (STAD), and Stomach and Esophageal carcinoma (STES).

We quantified the expression differences between normal and cancer samples in each tumor and then determined the significance of the expression differences. According to gene expression, the R package ESTIMATE was used to compute each patient’s stromal, immune, and ESTIMATE scores in each tumor. Moreover, the six types of immune cell infiltration scores of each patient in the tumor were re-evaluated using the Timer method. We used corr. test function of R software package “Psych” to calculate Pearson’s correlation coefficient between genes and immune infiltration score in each tumor to determine the immune infiltration score with significant correlation.

### 2.7. Statistical Analyses

The Kaplan–Meier approach was used to conduct the survival study. Using the “survivalROC” R package, we test the prediction performance of the risk model using time-dependent ROC analysis. Discontinuous data were displayed as numbers and percentages, whereas continuous data were provided in the form of mean ± standard deviation (SD). The student’s t-test was utilized for statistical analysis between two groups, and one-way ANOVA was chosen flexibly when three or more groups were determined. The *p* < 0.05 and FDR < 0.05 were indicated statistically significant differences. Statistical analyses were conducted via R. The whole process of data analysis is depicted in Figure 1B.

## 3. Results

### 3.1. Identification of Three Molecular Subgroups Using Lipid Metabolism Genes

**Figure 2:**
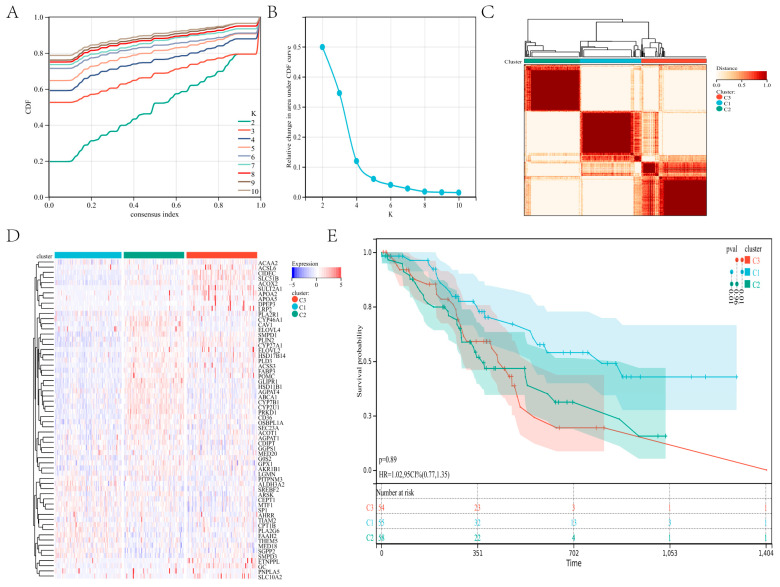
Consensus clustering: (**A**–**C**) The consensus cluster, K = 3, was determined to be the optimal value. (**D**) The heatmap in the three subtypes. (**E**) Survival curves for the three subtypes of patients.

Using the RNA expression data of 62 genes, we divided patients with gastric cancer into subgroups via the consensus clustering method. Using the empirical cumulative distribution function plot, we found the optimal clustering stability when K = 3 (Figure 2A–C; Figure S1A,B). The C1, C2, and C3 clusters each contained 55, 58, and 54 patients. A heatmap showed the level of lipid metabolism gene expression in the three subtypes, the expressions of lipid metabolism genes in clusters C2 and C3 were significantly higher than those in cluster C1 (Figure 2D). In addition, the survival analysis of the three clusters showed a significant difference between C1 and C2 (*p* = 0.01) and C1 and C3 (*p* = 0.01; Figure 2E). These findings indicated that the lipid metabolism genes divide individuals with gastric cancer into three distinct molecular subgroups with diverse survival rates, and the lower expression of the lipid metabolism cluster had higher survival.

### 3.2. Three Molecular Subgroups Displayed Distinct Immune Microenvironments

**Figure 3:**
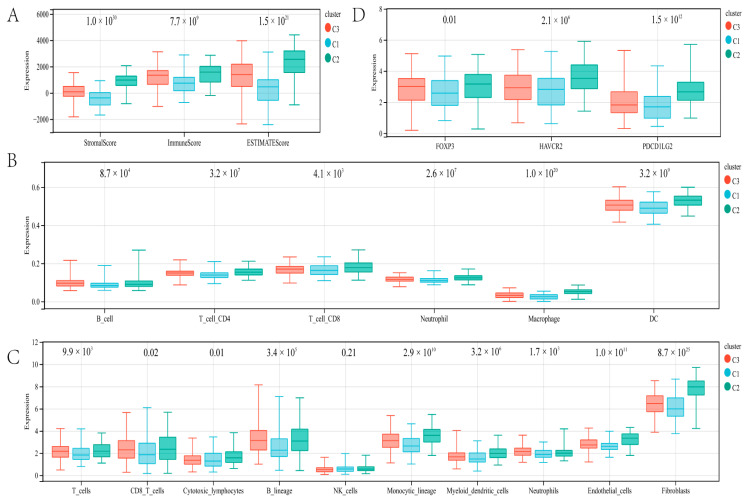
Immune landscape analysis: The immune microenvironment was evaluated by the ESTIMATE algorithm (**A**), TIMER algorithm (**B**), and MCPcounter algorithms (**C**). (**D**) Expression analysis of immune checkpoints.

Then, we used the immune method to identify the immune differences between the three molecular subgroups. Cluster C1 patients with gastric cancer had significantly lower stromal score (*p* = 1.0 × 10^30^), immune score (*p* = 7.7 × 10^9^), and ESTIMATE score (*p* = 1.5 × 10^21^) via ESTIMATE analysis (Figure 3A). In cluster C1, TIMER analysis showed that gastric cancer patients had significantly lower B cell (*p* = 8.7 × 10^4^), CD4 T cell (*p* = 3.2 × 10^7^), CD8 T cell (*p* = 4.1 × 10^3^), neutrophil cell (*p* = 2.6 × 10^7^), macrophage (*p* = 1.0 × 10^20^), and dendritic cell (*p* = 3.2 × 10^9^) compared with cluster C2 and cluster C3 (Figure 3B). In addition, MCPcounter algorithm indicated that the T cell (*p* = 9.9 × 10^3^), CD8 T cell (*p* = 0.02), B lineage (*p* = 3.4 × 10^5^), monocytic_lineage (*p* = 2.9 × 10^10^), myeloid_dendritic_cells (*p* = 3.2 × 10^6^), neutrophils (*p* = 1.7 × 10^3^), endothelial (*p* = 1.0 × 10^11^), and fibroblasts (*p* = 8.7 × 10^25^) in cluster C1 were significantly lower than cluster C2 and cluster C3. While there were lower cytotoxic lymphocytes in cluster C2, there were more in cluster C3 (*p* = 7.3 × 10^3^), and no statistical significance was detected concerning NK cells (*p* = 0.21; Figure 3C). In addition, we investigated several immune checkpoint genes. Compared with cluster C2 and C3, *FOXP3* (*p* = 0.01), *HAVCR2* (*p* = 2.1 × 10^6^), and *PDCD1LG2* (*PD-L2*; *p* = 1.5 × 10^12^) were lowly expressed in cluster C1 (Figure 3D). These findings indicate that the immune microenvironment varied significantly between the three molecular subgroups.

### 3.3. DEG and Functional Analyses

**Figure 4:**
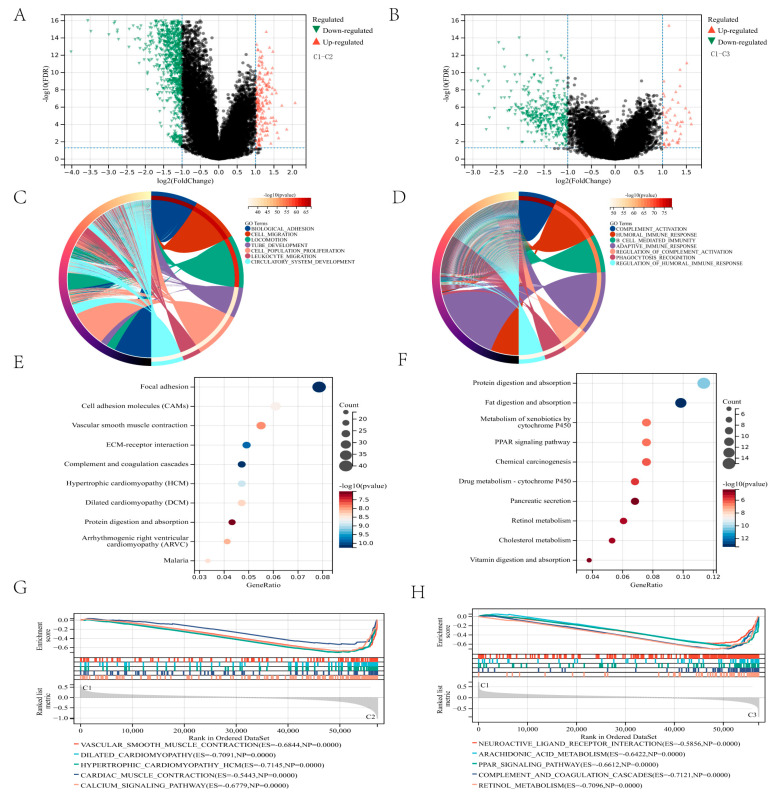
DEGs and functional enrichment analysis: (**A**,**B**) The volcano plot shows the DEGs between the two groups. (**C**,**D**) The circle plot illustrates the two groups’ biological processes enriched by GO analysis. (**E**,**F**) Bubble diagram illustrating the signaling pathways enriched by KEGG analysis between the two groups. (**G**,**H**) Plots visualize the results enriched by GSEA between the two groups.

We found DEGs between the three clusters and conducted functional enrichment studies to investigate the underlying signaling pathways. The significance threshold was FDR < 0.05, and the difference between the two groups was twofold. Compared clusters C1 and C2 amount to a total of 1274 differential genes were identified, 1096 genes were down-regulated, and 178 genes were up-regulated (Figure 4A). Compared clusters C1 and C3 amount to a total of 416 differential genes were identified, 366 genes were down-regulated, and 50 genes were up-regulated (Figure 4B). The expression level of lipid metabolism genes between cluster C1 and cluster C2, cluster C1, and cluster C3 was visualized through the heatmap (Figure S2A,B). GO enrichment analysis revealed that the DEGs between clusters C1 and C2 were enriched in cell migration, locomotion, and biological adhesion (Figure 4C). DEGs between clusters C1 and C3 were enriched in immune-related biological processes, including humoral immune response, B cell-mediated immunity, adaptive immune response, complement activation, and phagocytosis (Figure 4D). Additionally, several essential molecular functions and cellular components were enriched (Figure S3A–D).

KEGG analysis found several critical pathways linked with focal adhesion, complement, and coagulation cascades between cluster C1 and cluster C2 (Figure 4E) and several essential pathways linked with fat and protein digestion and absorption, retinol metabolism, and cholesterol metabolism between cluster C1 and cluster C3 (Figure 4F).

GSEA analyses were used to find the differential expression of the pathways in the three clusters further to reveal the relationship with the prognosis of gastric cancer. Compared to cluster C2, GSEA analysis revealed that vascular smooth muscle contraction and calcium signaling pathway are lowly expressed in cluster C1 (Figure 4G). Compared to cluster C3, GSEA analysis revealed arachidonic acid metabolism, complement and coagulation cascades, and retinol metabolism lowly in cluster C1 (Figure 4H). These results suggest a correlation between lipid metabolism gene expression, cell migration, and overexpression of immune response, which may contribute to the poor prognosis of patients with gastric cancer.

### 3.4. Development of a Risk Model Using Lipid Metabolism Genes in the Training Cohort

**Figure 5:**
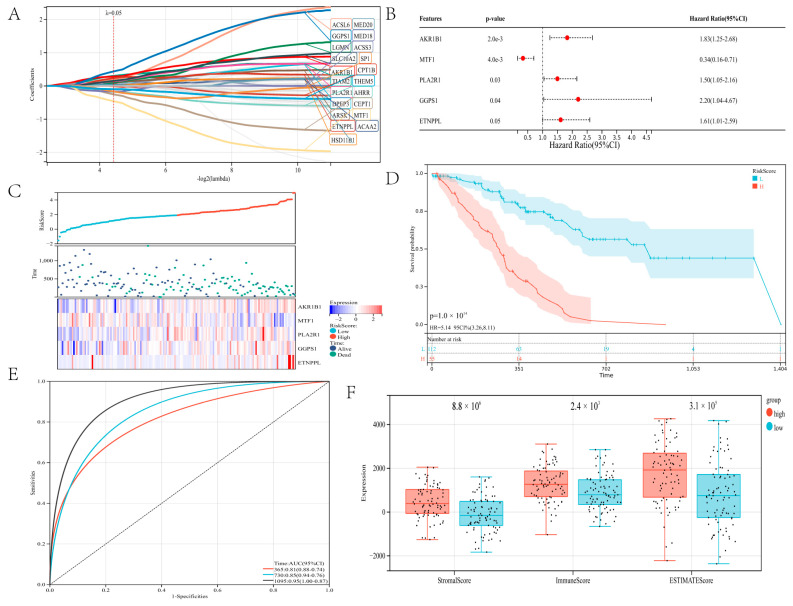
Structure a risk model: (**A**) The LASSO analysis. (**B**) A forest plot of five genes. (**C**) Shows the survival status of stomach cancer patients in the high-risk and low-risk groups and the expression of the five genes. (**D**) Survival curve of gastric cancer patients. (**E**) ROC curve depends on time for the risk model. (**F**) The ESTIMATE algorithm evaluated the immune scores of the two groups of gastric cancer patients.

The predictive efficacy of lipid metabolism genes in gastric cancer was assessed using a risk model. Twenty-one candidate genes for constructing a risk model were screened using LASSO analysis with a minimum lambda value (Figure 5A; Figure S4). The risk model was constructed using *AKR1B1*, *MTF1*, *PLA2R1*, *GGPS1*, and *ETNPPL* identified by LASSO and multivariate Cox analyses. These five genes are risk genes. *AKR1B1*, *PLA2R1*, *GGPS1*, and *ETNPPL* have hazard ratios exceeding one, while MTF1 has a 0.34 hazard ratio (Figure 5B). Kaplan–Meier analysis revealed that the five genes were independent prognostic indicators for patients with gastric cancer (Figure S5A–E). The built-risk model divided gastric cancer patients successfully into high-risk and low-risk groups. The expression of *AKR1B1*, *PLA2R1*, *GGPS1*, and *ETNPPL* genes tended to be higher in the high-risk group, while the expression of *MTF1* tended to be higher in the low-risk group (Figure 5C). Regarding overall survival, patients in the low-risk group performed better than those in the high-risk group (*p* = 1.0 × 10^14^; Figure 5D).

According to a time-dependent ROC analysis, the established risk model displayed exact prediction capability over three years. The AUC of the ROC curve for 365-day, 730-day, and 1095-day was 0.81, 0.85, and 0.95, respectively (Figure 5E). We ranked the risk scores from highest to lowest and divided them into two groups on average. The immune microenvironment of the two groups was evaluated using the ESTIMATE method. Compared to the high-risk group, the stromal score (*p* = 8.8 × 10^6^), immune score (*p* = 2.4 × 10^3^), and ESTIMATE score (*p* = 3.1 × 10^5^) were considerably lower in the low-risk group (Figure 5F). The higher the risk score, the higher the immune score. A positive correlation between risk score and the immune score has been reported in the literature in some tumors [33]. It has been reported that immune score is a strong prognostic factor for overall survival [34]. These results indicate that the developed risk model was fully capable of predicting the prognosis of patients with gastric cancer, and it was strongly related to TIME in gastric carcinoma.

### 3.5. Validation of Risk Model Independence

**Figure 6:**
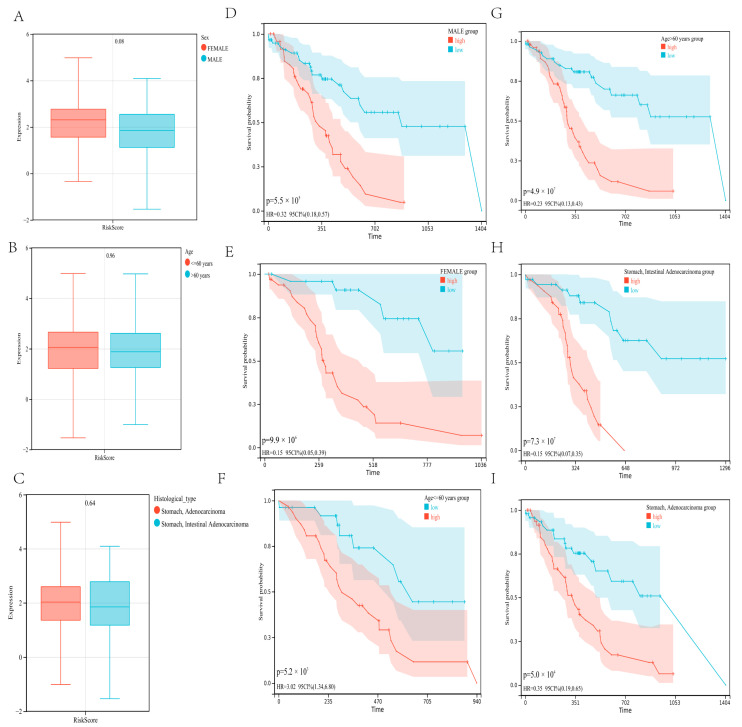
Correlation between risk score and clinical characteristics (**A**–**C**). No significant difference was determined in patients with different genders (**A**), ages (**B**), and histological types (**C**). Independence analysis of the risk model (**D**–**I**). The survival curve was regrouped by genders (**D**,**E**), ages (**F**,**G**), and histological types (**H**,**I**).

We also looked at the relationship between the risk score and clinical characteristics and used subgroup analysis and regression analyses to assess the developed risk model’s independence. There were no noteworthy differences among patients of different genders (*p* = 0.08), ages (*p* = 0.96), and histological types (*p* = 0.64). In terms of risk scores, there was no association between risk scores and sex, age, and histological types (Figure 6A–C). In addition, when the patients were regrouped by gender (Figure 6D,E), age (Figure 6F,G), and histological type (Figure 6H,I), the risk model continued to demonstrate strong prediction accuracy.

### 3.6. Risk Model Was Associated with TIME and Prognosis of Gastric Carcinoma in the Validation Cohort

**Figure 7:**
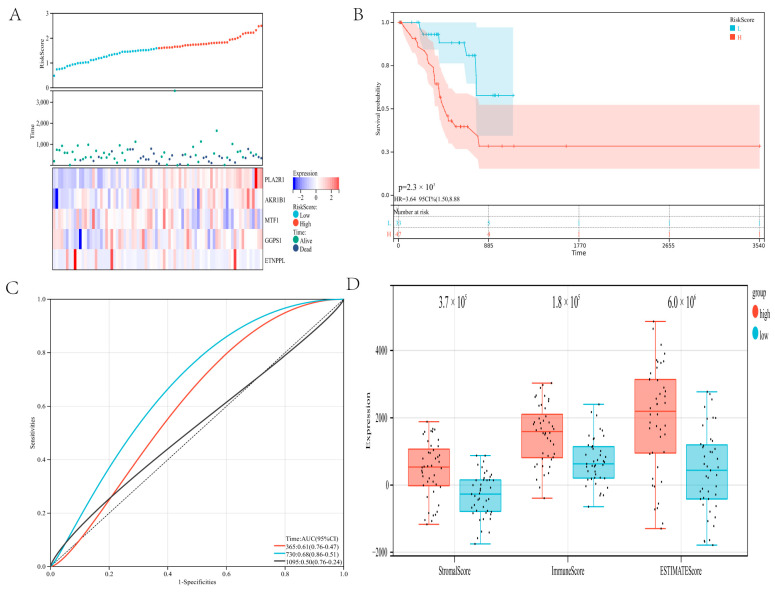
Validate the risk model: (**A**) Show survival status and the expression of five genes in the validation cohort. (**B**) In the verification cohort, the survival curves of patients in the high-risk and low-risk groups. (**C**) ROC curve of the risk model in the verification cohort. (**D**) The ESTIMATE algorithm evaluated the immune scores of the two groups of gastric cancer patients in the verification cohort.

The constructed prognostic risk model is further verified in the validation cohort. The gastric cancer patients in the validation cohort were stratified into a high-risk group and a low-risk group using the previous method. The expression of the five genes was shown through a heatmap (Figure 7A). The analysis of survival revealed that high-risk patients had a worse prognosis (*p* = 2.3 × 10^3^; Figure 7B). ROC analysis revealed that the risk model provided the most accurate prediction of 730-day survival (Figure 7C). The ROC curve of the risk model in the global set leads to the same conclusion (Figure S6).

In addition, we investigated the relationship between the risk model and the immune microenvironment. The low-risk group had a substantially lower stromal score (*p* = 3.7 × 10^5^), immune score (*p* = 1.8 × 10^5^), and ESTIMATE score (*p* = 6.0 × 10^6^) than the high-risk group (Figure 7D). Immune scores in the validation set are comparable to those in the training set. In the validation cohort, these results revealed that the developed risk model was connected with the immune microenvironment and prognosis for gastric cancer.

### 3.7. Development and Adjustment of an Integrated Monogram

**Figure 8:**
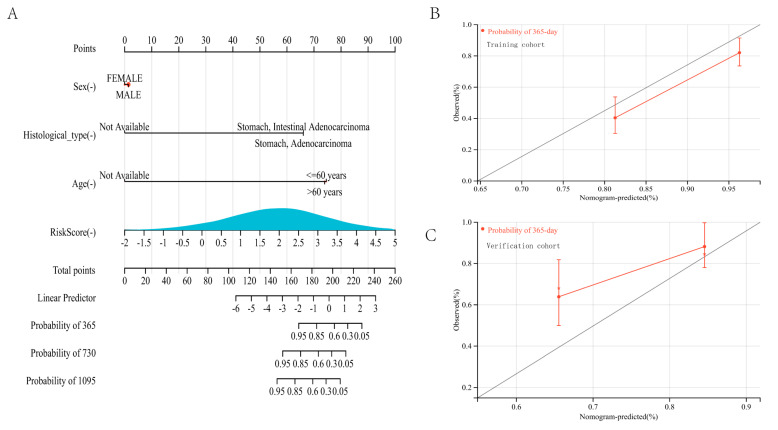
Construction and calibration of a nomogram: (**A**) Development and adjustment of a nomogram incorporating risk score and clinical characteristics. Adjustment of the nomogram at 365-day in the training cohort (**B**) and the verification cohort (**C**).

To better forecast the prognosis of gastric cancer patients, a nomogram was developed. A specific score was assigned via the created nomogram demonstrating the influence of risk score and clinical factors on the prognosis of gastric cancer patients (Figure 8A). Then, the nomogram was verified in the training and validation cohort. Regarding the model diagnostic of the nomogram, the calibration curve (Figure 8B,C) suggested sufficient precision. The C-index for the training cohort’s nomogram achieved 0.7439 (95%CI: 0.6887–0.7790). These findings demonstrated that the integrated nomogram could reliably predict the prognosis of gastric carcinoma patients. These results indicated that lipid metabolism dysregulation might cause cell migration and disorders of the immune system, culminating in a dismal prognosis. The built risk model using lipid metabolism genes correctly predicted the prognosis of patients with gastric cancer.

### 3.8. Differential Expression of Five Genes and Expression of Immune Infiltrates and Immune Cells in Gastrointestinal Cancer

*AKR1B1* was significantly up-regulated in ESCA (*p* = 8.4 × 10^4^), STES (*p* = 6.2 × 10^6^), STAD (*p* = 2.6 × 10^3^), and CHOL (*p* = 1.2 × 10^5^). *ETNPPL* was significantly down-regulated in ESCA (*p* = 3.8 × 10^3^), STES (*p* = 3.3 × 10^4^), STAD (*p* = 0.02), and CHOL (*p* = 1.2 × 10^5^). *GGPS1* was significantly upregulated in ESCA (*p* = 2.6 × 10^3^), STES (*p* = 3.2 × 10^11^), STAD (*p* = 3.0 × 10^9^), and CHOL (*p* = 2.3 × 10^9^). *MTF1* was significantly upregulated in STES (*p* = 0.01), STAD (*p* = 0.05), and CHOL (*p* = 5.3 × 10^6^), but not statistically significant in ESCA (*p* = 0.39). *PLA2R1* was significantly upregulated in ESCA (*p* = 0.02), STES (*p* = 5.4 × 10^3^), and CHOL (*p* = 8.2 × 10^5^) in three tumors, but not statistically significant in STAD (*p* = 0.11; Figure S7A–E).

**Figure 9:**
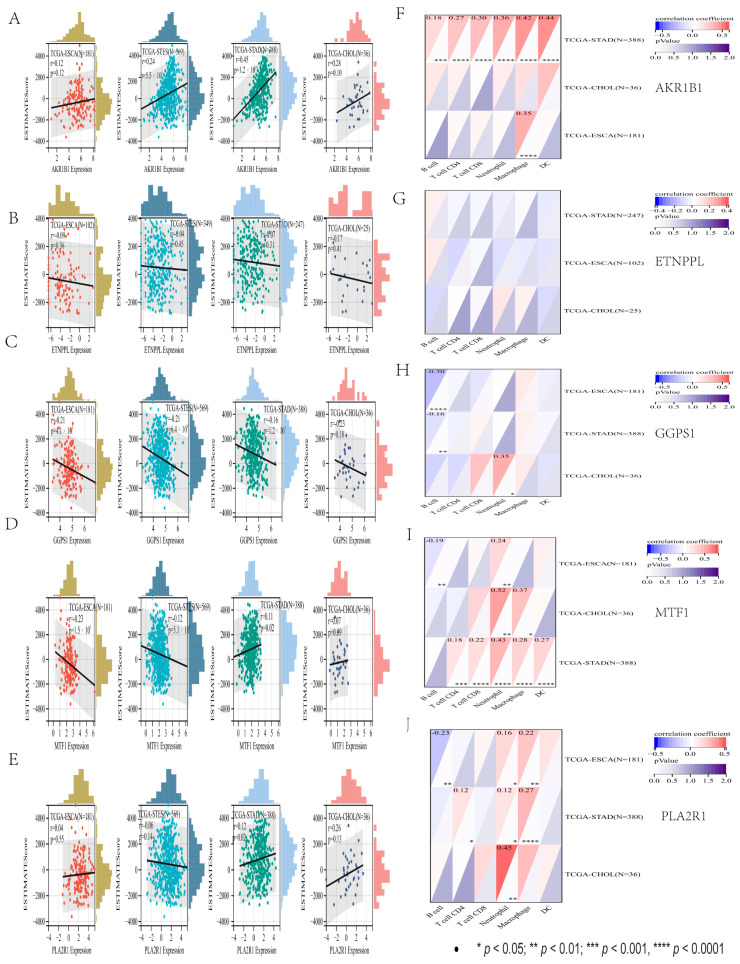
Five genes in gastrointestinal cancer differential and immune infiltration analysis. (**A**–**E**) The immune infiltration score. (**F**–**J**) The immune cells score.

We finally observed a significant positive correlation between *AKR1B1* expression and immune infiltration in two cancer species: STES (N = 569, R = 0.24, *p* = 5.5 × 10^9^), STAD (N = 388, R = 0.45, *p* = 1.2 × 10^20^). In the ESCA (N = 181, R = 0.12, *p* = 0.12), CHOL (N = 36, R = 0.28, *p* = 0.10) had no statistical significance. We observed that there was no statistical significance in *ETNPPL* expression and immune infiltration in four cancer species: STES (N = 349, R = 0.04, *p* = 0.45), STAD (N = 247, R = 0.07, *p* = 0.31), ESCA (N = 102, R = 0.09, *p* = 0.36), and CHOL (N = 25, R = 0.17, *p* = 0.41). We observed a significant negative correlation between *GGPS1* expression and immune infiltration in three cancer species: STES (N = 569, R = 0.21, *p* = 6.4 × 10^7^), STAD (N = 388, R = 0.16, *p* = 1.2 × 10^3^), and ESCA (N = 181, R = 0.21, *p* = 4.1 × 10^3^), but in CHOL (N = 36, R = 0.23, *p* = 0.18) there was no statistical significance. *MTF1* expression was significantly negatively correlated with immune infiltration: STES (N = 569, R = 0.12, *p* = 5.1 × 10^3^) and ESCA (N = 181, R = 0.23, *p* = 1.5 × 10^3^), but in STAD (N = 388, R = 0.11, *p* = 0.02) there was a significant positive correlation, CHOL (N = 36, R = 0.07, *p* = 0.69) showed no statistical significance. *PLA2R1* expression was significantly positively correlated with immune infiltration in STAD (N = 388, R = 0.12, *p* = 0.02), but STES (N = 569, R = −0.06, *p* = 0.14), ESCA (N = 181, R = −0.04, *p* = 0.55), and CHOL (N = 36, R = 0.26, *p* = 0.13) had no statistical significance (Figure 9A–E).

Through the TIMER algorithm, the expression of six types of immune cell infiltration scores of five genes in digestive tract cancer can be obtained. The *AKR1B1* gene in six kinds of immune cell infiltration results was extremely significant in gastric cancer. There was no statistical significance in six kinds of immune cell infiltration results of the *ETNPPL* gene in gastric cancer. Only the B cell score of the *GGPS1* gene was significant in gastric cancer. In the *MTF1* gene, except for the B cell score, there was no statistical significance in gastric cancer, and the scores of other the five immune cells were extremely significant. The B cells, CD8 T cell, and dendritic cell infiltration scores of the *PLA2R1* gene had no statistical significance in gastric cancer, while the infiltration scores of CD4 T cells and neutrophils were significant in gastric cancer, and macrophages were extremely significant (Figure 9F–J). These results showed that the differential expression of five genes in gastric cancer was consistent with the previous results, and the expression in other gastrointestinal cancers was further explored. Besides *ETNPPL*, the other four genes were also found to be closely related to the immune infiltration of gastric cancer, and the immune infiltration of five genes in other gastrointestinal cancers has a certain reference value for future studies.

## 4. Discussion

Gastric cancer is a classic heterogeneous cancer with molecular complexity and heterogeneity as one of the most aggressive tumors [35]. Gastric cancer heterogeneity has been highly complex with the advancement of technology in terms of genomic instability, differentially expressed genes, epigenetic heterogeneity, genetic variants, and protein heterogeneity. Nonetheless, novel biomarkers have helped create the molecular network of gastric cancer, improving our grasp of heterogeneity, and identifying more significant genetic subgroups associated with individual features [36].

We identified three molecular subtypes by intersecting genes related to survival and lipid metabolism. The immune assessment showed that the subgroup with high expression of lipid metabolism had a relatively higher immune status. Functional studies indicated lipid metabolism disorders associated with immune regulation and cell migration. In addition, we developed a predictive risk model based on lipid metabolism genes that accurately predicted the prognosis of patients with gastric cancer. Our findings may aid in developing gastric cancer-targeted therapy and assist doctors in making more sensible treatment decisions.

According to the gene expression matrix, consensus clustering proved a reliable method of classifying data into various subgroups. We identified three molecular groupings with significantly different overall survival using consensus clustering. The significance of lipid metabolism in gastric cancer was explored using immunological and functional analyses. The immune microenvironment is essential for patient prognosis because tumor growth is intimately linked to immune cells, and the abnormal metabolic condition of tumor cells might lead to variations in the immune microenvironment’s metabolism. The ESTIMATE was a novel algorithm for calculating the proportion of immune and stromal cells in tumors. According to our findings, the cluster with high expression of lipid metabolism had a relatively higher immune score. In addition, we utilized the TIMER and MCPcounter algorithm to evaluate the immunological state of the three molecular subtypes. In agreement with the ESTIMATE finding, the TIMER analysis showed that the quantity of six immune cells was considerably lower in cluster C1, indicating that immune processes are downregulated in cluster C1. The MCPcounter result revealed that patients in cluster C1 had a comparatively poor immunological state, confirming the ESTIMATE and TIMER findings. Since the relationship between lipid metabolism and immune responses in the tumor environment is unclear, we speculate that immune cells need a large number of fatty acids for energy when they compete with cancer cells, thus exhibiting high immune scores in high lipid metabolic clusters. Different immune-infiltrating cells can enhance or suppress antitumor immunity in TIM [37]. The phenotype of macrophages is closely related to the reprogramming of lipid metabolism in cancer cells. Macrophages in the clusters C2 and C3 are highly expressed, and M2 macrophages can enhance fatty acid oxidative phosphorylation to supply energy and promote the occurrence and development of tumors [38]. Cluster C1 with low expression of lipid metabolism had a low immune score. The effect of lipid accumulation caused by abnormal lipid metabolism on DCs in the TIME may also be partly responsible for the poor prognosis. Abnormal lipid accumulation inhibits the ability of DCs to promote antitumor T cells [39]. This is why downregulated lipid metabolism leads to lower immune scores and immune status. Moreover, since the metastatic potential of tumor cells is positively correlated with intracellular lipid storage, the accumulated lipids may contribute to poor prognosis by promoting metastasis. Immune checkpoint genes are highly expressed in clusters C2 and C3, promoting immune escape and poor prognosis.

Then, to understand the underlying biological mechanisms, functional studies were undertaken comparing the three clusters. Based on discovered DEGs, GO, and KEGG analyses revealed that immunological modulation and cell migration might modulate the role of lipid metabolism in gastric cancer development and progression. However, the precise association between lipid metabolism, immunological modulation, and cell migration remained unknown. As a result, we used GSEA analysis to acquire more about the underlying mechanisms. Compared to clusters C2 and C3, GSEA results revealed the comparatively low expression of vascular smooth muscle contraction, calcium signaling pathway, and lipid metabolism in cluster C1. These findings suggest that the immunological state, vascular smooth muscle contraction, and cell migration were linked to lipid metabolism.

By combining the initial findings, we can conclude that disturbance of lipid metabolism impacted the immune microenvironment and cell migration, resulting in a poor prognosis for gastric cancer. As previously reported, lipid metabolism reprogramming has been identified as a new characteristic of tumor aggressiveness. Lipid metabolic anomalies in tumors have received a lot of attention in recent years. Antitumor treatment that targets abnormal lipid metabolic pathways is a potential technique [40]. It is reported that some pathways, such as the PI3K pathway, participate in the progression of cancer via enhancing cell proliferation and angiogenesis, promoting cell migration and infiltration, and suppressing apoptosis [41]. The functional analysis in this paper further verifies this conclusion.

We built a predictive risk model using lipid metabolism genes and validated it in the verification cohort. This can confirm the impact of lipid metabolism disturbance on the immune microenvironment in patients with gastric cancer and assess the prognostic significance of lipid metabolism in patients with gastric cancer.

The five genes utilized to create the risk model are closely linked to tumor formation and development. PGF2α is the primary metabolite catalyzed by *AKR1B1*. PGF2α regulates the signaling pathway of tumor endothelial cells through the PGF2α receptor, thus regulating cell adhesion, invasion, and migration [42]. Pathways associated with *MTF1* include Metabolism and Regulation of cholesterol biosynthesis by SREBP (SREBF). SREBPs (SREBFs) respond to low cholesterol concentrations by transiting the nucleus and activating cholesterol and lipid biosynthesis genes. Pathways associated with *PLA2R1* include metabolism and acyl chain remodeling of PE [43]. The related pathways of *GGPS1* are metabolism and cholesterol biosynthesis I [44]. *ETNPPL* has associated pathways, including metabolism and glycerophospholipid biosynthesis, and it enables ethanolamine-phosphate phospho-lyase activity [45].

According to survival analyses, the constructed risk model had a powerful prediction performance for the survival of gastric cancer patients in both the training and verification cohorts. Furthermore, independence and subgroup analyses revealed that the risk model is able to estimate prognostic in gastric cancer patients independently of their gender, age, and histological type. A nomogram incorporating the risk score and clinical variables was developed and validated, demonstrating the significant predictive ability for survival. These findings supported the importance of lipid metabolism genes in prognosticating gastric cancer and the association between abnormal lipid metabolism and immune microenvironment disorder.

Despite numerous breakthroughs in surgical surgery and chemotherapy, the overall survival rate has remained unchanged. Patients with stomach cancer have a bad prognosis [46]. It is critical to create efficient strategies for classifying patients based on their risk scores and providing appropriate, tailored treatment. However, there have been several studies examining risk models of gastric cancer [47]. As compared to earlier research, ours had specific merits:

1. We looked at the lipid metabolism genes and used consensus clustering to identify three molecular groupings with significantly different prognoses and immunological states.
2. We delved into biological pathways based on clustering results and partially revealed the underlying mechanism.
3. We investigated whether lipid metabolism affects the immune microenvironment and prognosis.
4. We explored the differential expression and immune microenvironment of five essential genes in digestive cancer, which confirmed the rationale for using essential genes to build a predictive model.

Our findings provide valuable theoretical guidance for future gastric cancer research.

We found three molecular subtypes in our study: cluster C1, cluster C2, and cluster C3. Patients in cluster C1 with low expression of lipid metabolism had lower immunological scores. Lipid metabolism dysregulation was linked to a high immune status and cell migration. The prognosis of gastric cancer could be accurately predicted using a risk model. These findings suggested that the lipid metabolism landscape was linked to the immune microenvironment. When deciding on a treatment strategy for gastric cancer patients who could benefit from tailored treatment, it was worth paying close attention. In addition, after constructing the gastric cancer risk prognosis model, this study further explored the expression of critical genes in digestive tract cancer and immune infiltration, which verified the importance of critical genes and played some role in the study of biomarkers in the digestive tract cancer.

There are some imperfections in our study. For starters, we were unable to demonstrate the function of lipid metabolism genes in the progression of gastric carcinoma due to a lack of information regarding the patients’ advancement, such as tumor stages. Second, the outcomes of our bioinformatics study were not further confirmed through experimentation. Finally, instead of using our cohort, the data were obtained from several open databases. Because all of the patients in this study were chosen retrospectively, there is a risk of bias due to unbalanced clinicopathological characteristics and treatment heterogeneity. To confirm the predictive value of lipid metabolism genes in gastric cancer, more prospective studies are needed.

## 5. Conclusions

Finally, three molecular subtypes of gastric cancer were identified using consensus clustering. According to immunological and functional assessments, lipid metabolism disorders affect the immune system and cell migration, resulting in a poor prognosis. The connection between the immune microenvironment and lipid metabolism was investigated in this work along with the construction of a gastric cancer risk prognosis model. Preliminarily exploring the differential expression of five genes and the immune situation in gastrointestinal cancers further confirms the importance of these five lipid metabolism genes. Some contributions have been made in the discovery of biomarkers for gastrointestinal tumors. Our findings can help develop new targeted medications and risk stratifications for gastric cancer patients.

## Figures and Tables

**Table 1:** The genes used for constructing the risk model.

Gene Name	Full Name	Category	Function
*AKR1B1*	Aldo-Keto Reductase Family1 Member B	Protein Coding	*KR1B1* is an NADPH-dependent PGF2α synthase involved in arachidonic acid metabolism.
*MTF1*	Metal-regulated Transcription Factor 1	Protein Coding	Metabolism and regulation of cholesterol biosynthesis by SREBP (SREBF)
*PLA2R1*	Phospholipase A2 Receptor 1	Protein Coding	Metabolism and acyl chain remodeling of phosphatidyl ethanolamine (PE).
*GGPS1*	Geranylgeranyl Diphosphate Synthase 1	Protein Coding	Metabolism and cholesterol biosynthesis I
*ETNPP*	Ethanolamine-Phosphate Phospho-Lyase	Protein Coding	Metabolism and glycerophospholipid biosynthesis, and enhancement enables ethanolamine-phosphate phospho-lyase activity

## Data Availability

The data sets used in this study are all available in online public databases.

## Supplementary Materials

The following supporting information can be downloaded at: https://www.mdpi.com/article/10.3390/genes13091581/s1, Figure S1: Consensus clustering of stomach cancer patients; Figure S2: The expression level of lipid-metabolism genes between two groups; Figure S3: GO analysis of the DEGs between the two groups; Figure S4: Lambdas values analyzed by LASSO; Figure S5: Survival analysis of the five genes; Figure S6: The ROC curve of the complete set risk model.; Figure S7: Five genes were differentially expressed in gastrointestinal carcinoma.
