# Supplementary material for: Bioinformatics Analysis and Structure of Gastric Cancer Prognosis Model Based on Lipid Metabolism and Immune Microenvironment
Source: Genes (Basel). 2022 Sep 3;13(9):1581. doi: 10.3390/genes13091581 (PMC9498347; doi:10.3390/genes13091581)
Supplement: Supplementary file 1 [file genes-13-01581-s001.zip › genes-1812510-supplementary.pdf]

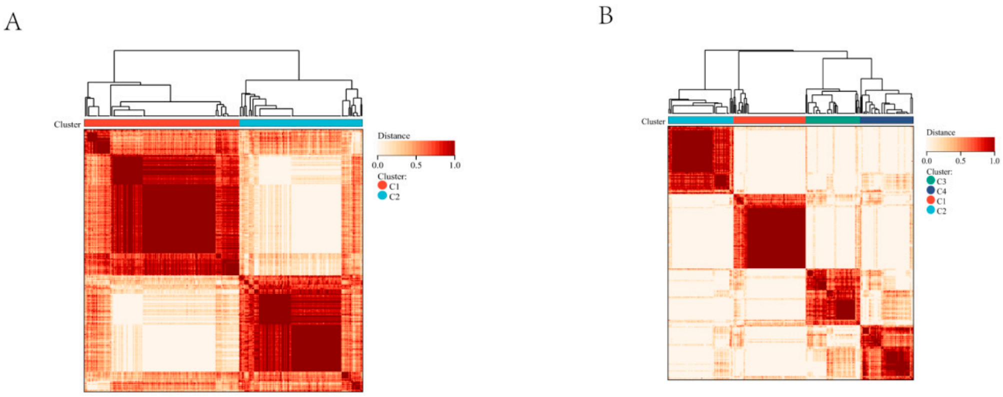

Supplementary Figure S1. Consensus clustering of stomach cancer patients.K = 2 (A), K=3 (B).

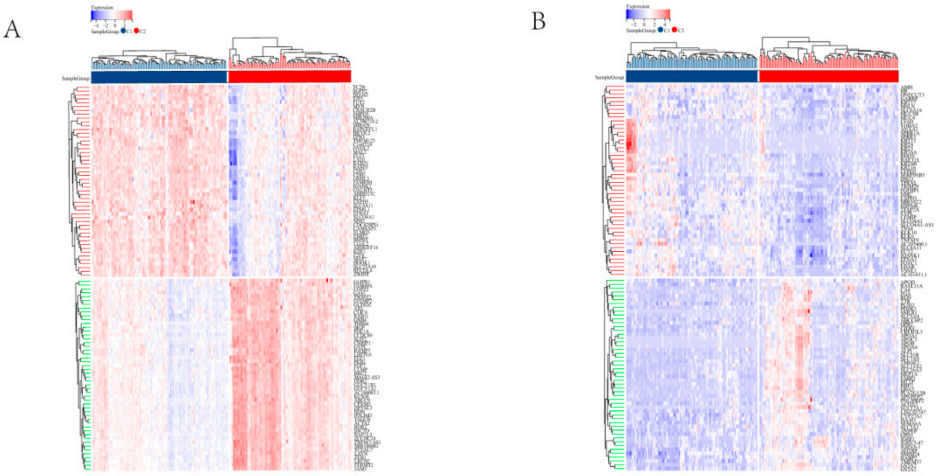

Supplementary Figure S2. (A, B) The expression level of lipid-metabolism genes between two groups.

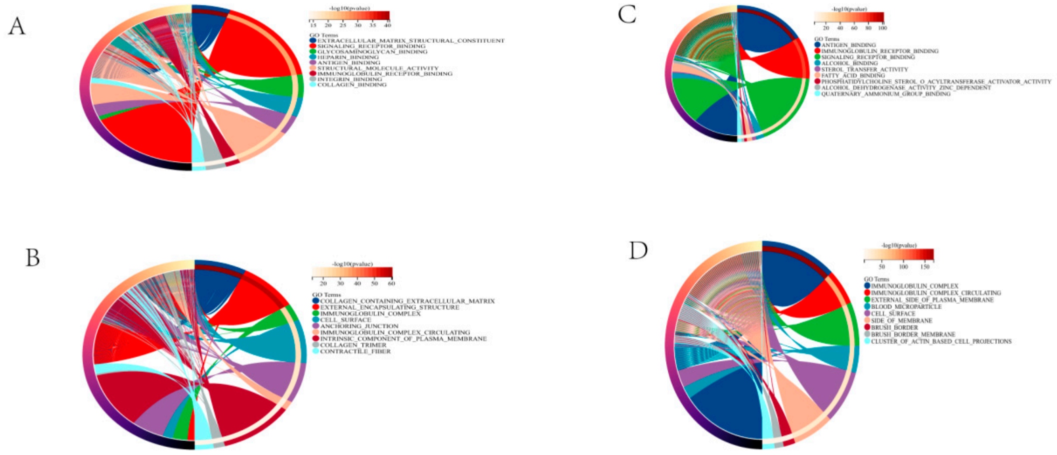

Supplementary Figure S3. GO analysis of the DEGs between the two groups. (A, B) Molecular function enrichment analysis of the differentially expressed genes. (C, D) Cellular components enrichment analysis of the differentially expressed genes.

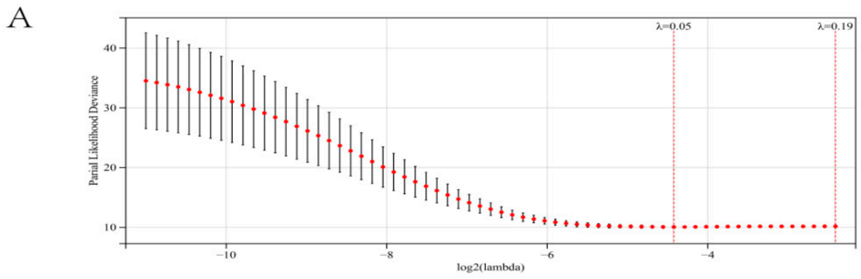

Supplementary Figure S4. Lambdas values analyzed by LASSO.

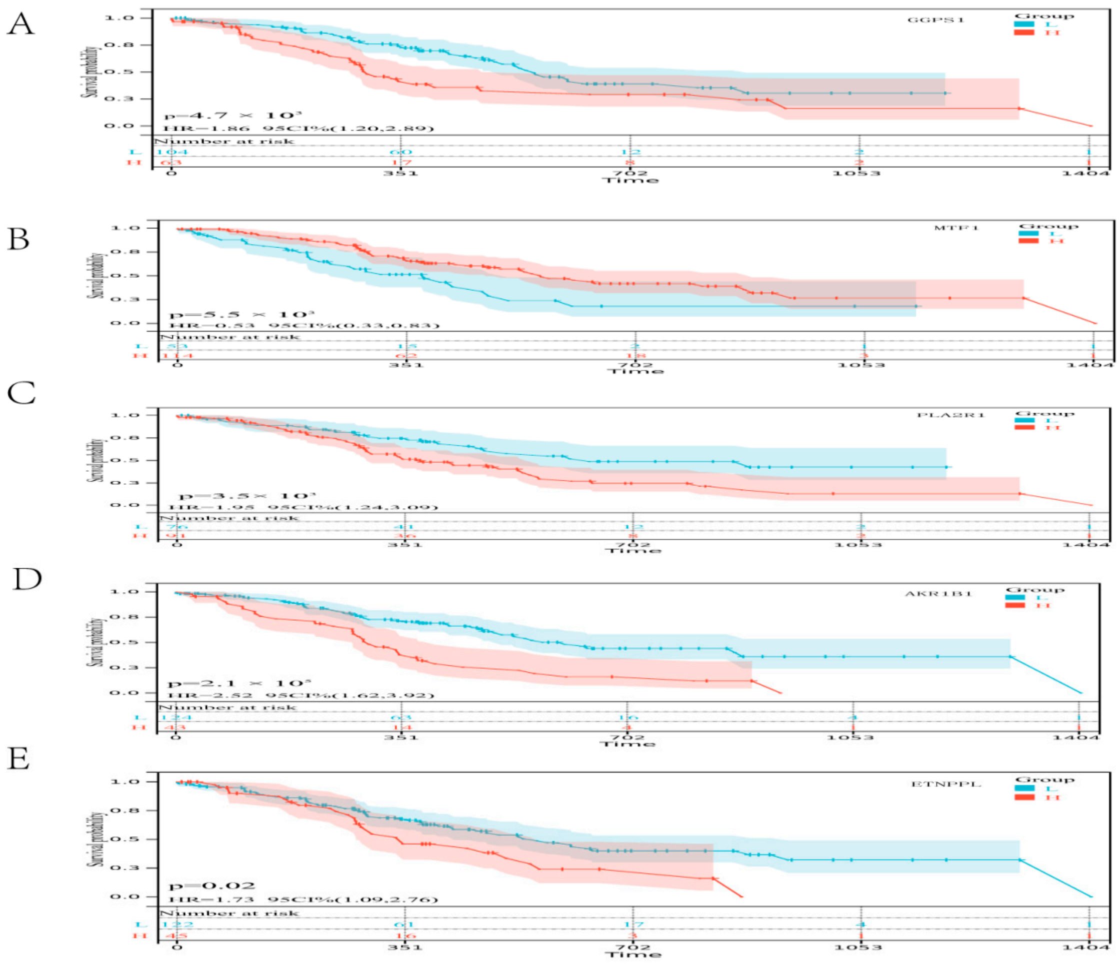

Supplementary Figure S5. (A-E) Survival analysis of the five genes. All *AKR1B1*, *MTF1*, *PLA2R1*, *GGPS1*, and *ETNPPL* were independently prognostic genes in stomach cancer.

A

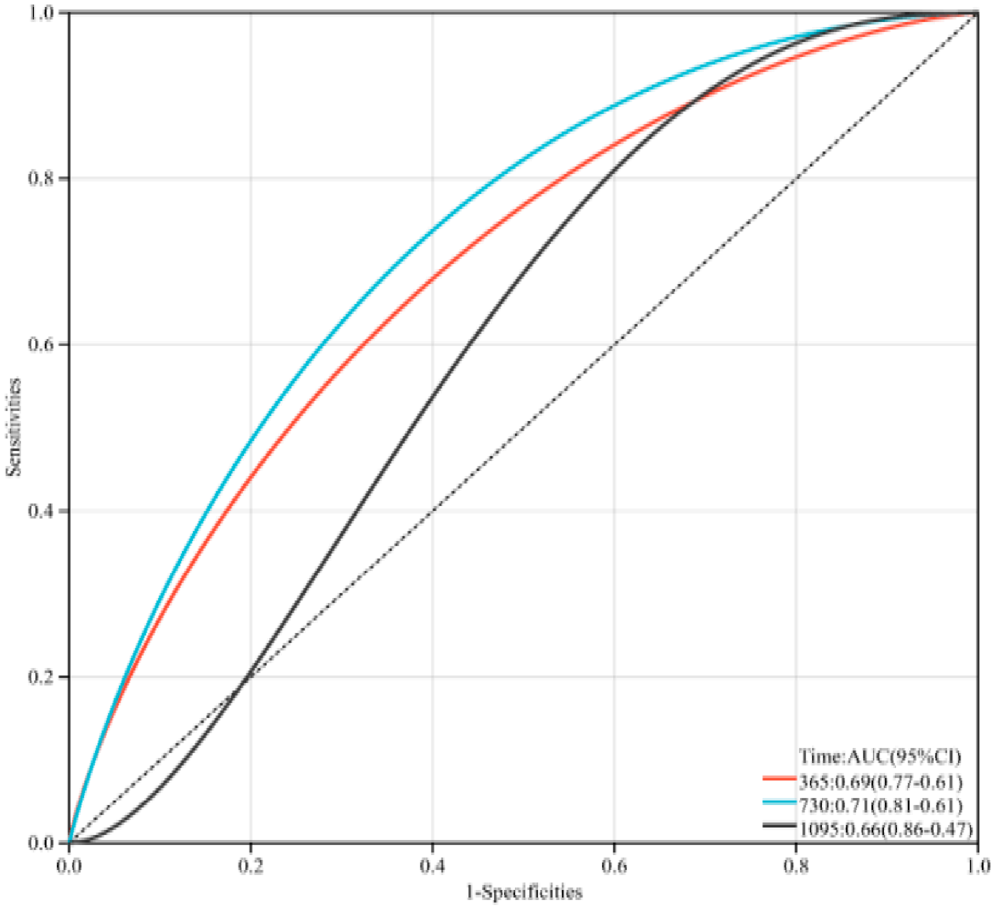

Supplementary Figure S6. The ROC curve of the complete set risk model.

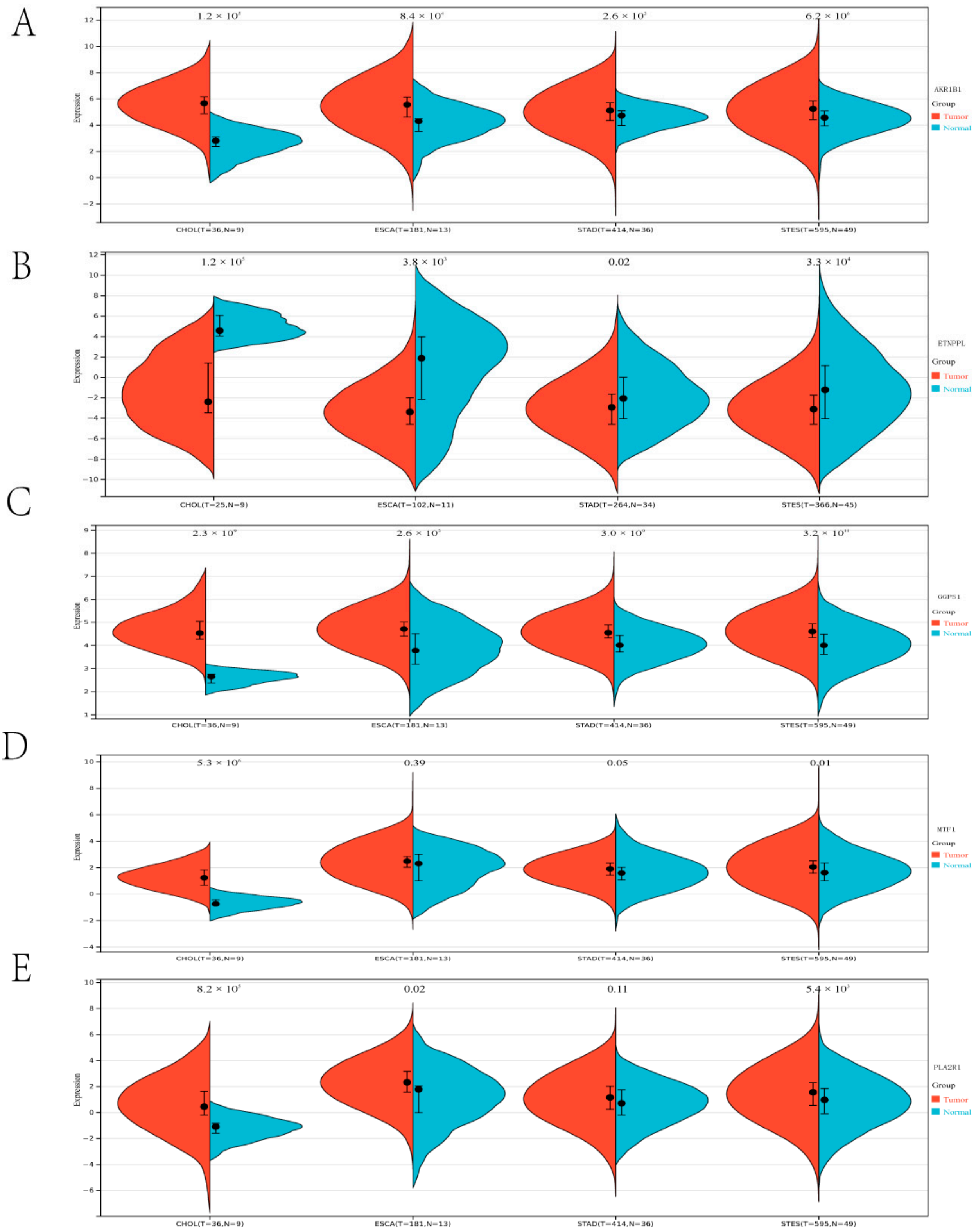

Supplementary Figure S7. (A-E) Five genes were differentially expressed in gastrointestinal carcinoma.
